# Commentary: The origins of intellectual disability

**DOI:** 10.1111/jcpp.13591

**Published:** 2022-02-18

**Authors:** Robert Plomin

**Affiliations:** ^1^ King’s College London London UK

## Abstract

Despite the importance and prevalence of intellectual disability (ID), its origins have not been well understood until now. Lichtenstein et al. report in this issue findings from a population‐based sample four times larger than all previous family studies of ID put together (Lichtenstein et al., 2022). From more than four million people, 37,787 individuals were identified with ID. Relative risks (RRs) are reported for relatives of ID probands (55,000 first‐degree, 55,000 second‐degree, and 170,000 third‐degree) as compared with matched relatives of individuals without ID. These relatives plus 400 pairs of twins in which at least one twin was diagnosed with ID yield an astonishing estimate of 95% heritability with no evidence for shared environmental influence in their model. Another important finding is that maternal half‐siblings of ID individuals were at greater risk than paternal half‐siblings, a maternal effect that could indicate X‐chromosome linkage. Finally, profound and severe ID is etiologically distinct from the normal distribution, due in part to noninherited (de novo) genetic mutations and chromosomal abnormalities. However, 90% of individuals with ID are moderate or mild, and these represent the low end of the normal distribution of genetic influence on cognitive ability, which has important implications for DNA research on ID.

## Introduction

The paper by Lichtenstein et al. in this issue is certain to become a classic in research on intellectual disability (ID) (Lichtenstein et al., 2022). The sampling frame is the total Swedish population of over four million people with links to health and education registers. From this cohort, ID was reported for 37,787 individuals. This ID prevalence of 0.9% matches the prevalence reported in a meta‐analysis of 52 studies of ID (Maulik, Mascarenhas, Mathers, Dua, & Saxena, 2011). Sweden’s system of unique personal identification numbers enables identification of family members, which makes this not only the first population‐based study of familial risk for ID but also the first study to calculate relative risk (RR) by comparing relatives of ID probands with matched relatives of unaffected individuals.

The sample of ID probands is four times larger than all previous family studies of ID put together. In earlier studies, 75% of the relatives were full siblings, whereas the Swedish study includes not only 46,174 full siblings but also large samples of all other first‐degree relatives (5125 parents, 3680 children), second‐degree relatives (12,074 maternal half‐siblings, 11,981 paternal half‐siblings, 20,911 nephews/nieces, 10,712 uncles/aunts), and third‐degree relatives (169,432 first cousins). And a population comparison sample of four million relatives of individuals without ID!

The study provides a rich harvest of results about family risk, genetic influence, environmental effects, sex differences, and whether ID is best viewed as a disorder or the lower end of the normal distribution. One unavoidable limitation is that these results describe a particular population (Sweden) at a particular time (1973‐2013 births).

## Family risk

The RR estimates are useful clinically to predict family risk. For example, full siblings of ID probands are eight times more likely to receive an ID diagnosis than siblings of individuals without an ID diagnosis (RR = 8.4). For children of ID probands, the RR is 14.8. The RR drops to 3.5 for second‐degree relatives. The RR falls further to 2.0 for third‐degree relatives.

## Heritability

The overall pattern of resemblance for first‐, second‐, and third‐degree relatives is consistent with genetic influence, but it could also be explained by shared family environment that decreases with decreasing relatedness. The twin design can disentangle these competing hypotheses of nature and nurture. The population sweep picked up the expected number of twins – 116 pairs of MZ twins and 284 pairs of DZ twins in which at least one member of the twin pair had received an ID diagnosis. For MZ twins, the probandwise twin concordance was 73%. In contrast, the DZ twin concordance was only 9%, similar to the concordance for full siblings. These results strongly support the genetic hypothesis. MZ and DZ tetrachoric correlations derived from the liability‐threshold model were 0.97 and 0.43, respectively, suggesting a heritability of liability of almost 100%.

A liability‐threshold model‐fitting analysis of these twin data together with all the other relatives yielded a heritability estimate of 95%, which makes this the highest estimate of heritability for a behavioral disorder. However, three points should be noted about this astonishingly high heritability estimate. First, this estimate depends on the high MZ concordance of 73%, much more than twice as high as the DZ concordance of 9%. This pattern of twin concordance is also seen for autism. For narrowly defined autism, MZ and DZ concordances are 76% and 0%, respectively; for broader autistic spectrum disorder, concordances are 88% and 31% (Ronald & Hoekstra, 2011). It is not clear why MZ concordances are so high relative to DZ concordances. The phenomenon is unlikely to be due to prenatal effects because MZ twins generally experience greater differences prenatally as compared with DZ twins (Knopik, Neiderhiser, DeFries, & Plomin, 2017). One possibility is higher‐order nonadditive genetic effects, called epistasis. MZ twins are identical in terms of inherited differences in DNA sequence and are thus identical in terms of nonadditive genetic effects. DZ twins correlate 0.50 for additive genetic effects, in which the effects of each DNA variant add up across the genome, but correlate near zero for nonadditive effects. However, this hypothesis cannot explain the smooth reduction in familial resemblance from first‐degree to second‐degree to third‐degree relatives. Perhaps different factors are responsible for the high MZ concordance and concordance for other relatives.

Second, although the heritability estimate of 95% is derived from a model‐fitting analysis based on all the family data, the model hinges substantially on the twin sample. Although the sample of 116 MZ and 284 DZ twin pairs is large for research on ID, it is nonetheless modest in terms of the power of its point estimate of heritability. Another twin study of mild ID, also from Sweden, used a different technique to estimate heritability as 46% (95% CCI: 0.19–0.41) based on 79 pairs of MZ twins and 123 pairs of DZ twins (Reichenberg et al., 2016).

Third, it should be noted that 95% is not the heritability of ID *as dichotomously diagnosed*. It is the heritability of a hypothetical construct of a continuous liability in which ID appears above a certain threshold, in this case, the 0.9% prevalence. The liability‐threshold model‐fitting analysis begins by estimating tetrachoric twin correlations derived from dichotomous diagnoses of ID. In contrast, the heritability of ID *as diagnosed* cannot exceed the MZ twin concordance of 73% because MZ twins are identical in terms of inherited differences in DNA sequence.

Nevertheless, regardless of whether heritability is viewed in relation to liability (95%) or MZ concordance (73%), the data provide strong evidence for the importance of inherited DNA differences in the etiology of ID.

## Maternal effects on inheritance patterns

The second‐degree relatives include two groups of half‐siblings: those related through their mother or their father. The relative risks for ID for the two groups are 4.6 and 2.9, respectively, a statistically significant difference. This maternal effect could be a shared environmental effect, although this would seem unlikely if the twin study estimate of zero shared environmental influence is to be believed. This remains an open question because the other Swedish twin study of mild ID mentioned earlier yielded a shared environmental estimate of 30% (Reichenberg et al., 2016).

A prenatal hypothesis would nonetheless be possible because half‐siblings related through their mother share the same womb at different times. Such a prenatal effect would not necessarily be seen for twins because both MZ and DZ twins share the same womb at the same time. In addition, there is some evidence that MZ twins experience greater differences prenatally than DZ twins. For example, MZ twins show greater birth weight differences than DZ twins (Knopik et al., 2017). Such a differential prenatal effect would diminish evidence for shared environment in twin studies. One point against a prenatal hypothesis is that the Lichtenstein et al. found no support for the hypothesis that adverse birth effects cause ID. However, this finding goes against a substantial literature suggesting an environmental basis for ID such as fetal alcohol syndrome, congenital infections, and pregnancy complications (Reichenberg et al., 2016).

In line with a genetic explanation, the relative risk for maternal half‐siblings (RR = 4.6) is half the relative risk of full siblings (RR = 8.4), even though both types of siblings began life in the same womb. Another genetic contributor to the low relative risk for paternal half‐siblings (RR = 2.9) is mistaken paternity. A specific genetic hypothesis is that recessive DNA variants on the X chromosome are involved, as explained in the following section.

## Why are more males diagnosed with ID?

A puzzle in child psychiatry is why the prevalence of several childhood disorders, including ID, is much greater in males than females. In the Lichtenstein et al. study, 59% of the ID probands were male and 41% female. They also showed that for ID probands, risk was greater for male relatives than for female relatives.

One genetic hypothesis is that recessive alleles on the X chromosome are responsible for such sex differences. Females have two X chromosomes, one from the mother and one from the father; males receive one X chromosome from their mother and a small Y chromosome from their father. If recessive alleles on the X chromosome are associated with ID, males will manifest the disorder more often because with only one X chromosome, they express recessive alleles on the X chromosome. Females only manifest the disorder if they inherit the recessive allele on both of their X chromosomes. Thus, the ID risk for half‐siblings related through their mother would be expected to be greater than for half‐siblings related through their father, which is what was found in the Lichtenstein et al. study (RR = 4.6 vs 2.9). This effect would be expected to be greater for male half‐siblings than for female half‐siblings, but the authors did not report this comparison.

There is another more direct test of the X‐chromosome linkage hypothesis which the authors did not report: comparing father‐son ID risk to risk for the three other parent‐offspring sex comparisons. Because sons only inherit the Y chromosome from their fathers, if DNA differences on the X‐chromosome impact ID, father‐son ID risk would be expected to be lower than ID risk for the other parent‐offspring combinations.

## Is ID the lower end of the normal distribution?

Common disorders are generally the quantitative extreme of the same genetic and environmental factors that affect variability throughout the normal distribution (Plomin, Haworth, & Davis, 2009). In other words, there are no etiologically distinct common disorders, just continuous distributions. However, the Lichtenstein et al. paper supports previous findings suggesting that severe/profound ID is etiologically distinct, often caused by noninherited (de novo) mutations and chromosomal abnormalities (Reichenberg et al., 2016). Nonetheless, only 10% of ID probands were in the profound or severe categories; the other 90% represent the low end of the normal distribution of genetic influence on cognitive ability.

## DNA

Molecular genetic research on ID has found some rare single‐gene mutations and chromosomal anomalies responsible for severe ID (Veltman & Brunner, 2012). However, the finding that 90% of individuals diagnosed with ID represent the low end of the normal distribution of cognitive ability has implications for molecular genetic research. It suggests that ID, like other common disorders, is best conceptualized as the quantitative extreme of a highly polygenic liability, which means that many thousands of tiny inherited DNA differences contribute to its heritability.

To identify DNA differences associated with ID, it is not necessary to conduct case‐control genome‐wide association (GWA) analyses comparing ID probands to controls. The implication of finding that 90% of individuals with ID represent the lower end of the normal distribution is that GWA studies of normal variability in cognitive ability will yield polygenic scores that predict ID just as well as they predict high cognitive ability and everyone in between. Polygenic scores can already predict more than 10% of the variance of cognitive ability (von Stumm & Plomin, 2021). These polygenic scores could help distinguish ID due to de novo mutations from ID at the low end of the normal distribution because individuals with ID due to de novo mutations are likely to have higher polygenic scores than individuals with common ID.

Polygenic scores are unique predictors because they can predict from early in life and do not change, which means that they can be used to predict ID before intelligence can be assessed. This makes them especially valuable, for example, in helping to identify infants at risk for later learning difficulties so that they receive the support they need early on. Polygenic scores are also unique in that they can predict differences within a family. Even if ID was 100% heritable, siblings would still differ substantially because they correlate only 0.5 genetically.

In summary, the implications of the Lichtenstein et al. study will reverberate for a long time to come in research, in clinics, and, eventually, in society.
